# Ethnically Disparate Disease Progression and Outcomes among Acute Rheumatic Fever Patients in New Zealand, 1989–2015

**DOI:** 10.3201/eid2707.203045

**Published:** 2021-07

**Authors:** Jane Oliver, Oliver Robertson, Jane Zhang, Brooke L. Marsters, Dianne Sika-Paotonu, Susan Jack, Julie Bennett, Deborah A. Williamson, Nigel Wilson, Nevil Pierse, Michael G. Baker

**Affiliations:** Murdoch Children’s Research Institute, Melbourne, Victoria, Australia (J. Oliver);; University of Otago, Wellington, New Zealand (J. Oliver, O. Robertson, J. Zhang, B.L. Marsters, S. Jack, J. Bennett, N. Pierse, M.G. Baker);; University of Melbourne, Melbourne (J. Oliver, D.A. Williamson);; University of Otago, Wellington (D. Sika-Paotonu);; Victoria University of Wellington, Wellington (D. Sika-Paotonu); Southern District Health Board, Dunedin (S. Jack);; Starship Child Health, Auckland (N. Wilson)

**Keywords:** Epidemiology, acute rheumatic fever, rheumatic heart disease, disease burden, New Zealand, public health, group A Streptococcus, Māori, Pacific Islander, bacteria, streptococci

## Abstract

We investigated outcomes for patients born after 1983 and hospitalized with initial acute rheumatic fever (ARF) in New Zealand during 1989–2012. We linked ARF progression outcome data (recurrent hospitalization for ARF, hospitalization for rheumatic heart disease [RHD], and death from circulatory causes) for 1989–2015. Retrospective analysis identified initial RHD patients <40 years of age who were hospitalized during 2010–2015 and previously hospitalized for ARF. Most (86.4%) of the 2,182 initial ARF patients did not experience disease progression by the end of 2015. Progression probability after 26.8 years of theoretical follow-up was 24.0%; probability of death, 1.0%. Progression was more rapid and ≈2 times more likely for indigenous Māori or Pacific Islander patients. Of 435 initial RHD patients, 82.2% had not been previously hospitalized for ARF. This young cohort demonstrated low mortality rates but considerable illness, especially among underserved populations. A national patient register could help monitor, prevent, and reduce ARF progression.

Acute rheumatic fever (ARF) is a rare inflammatory condition triggered in response to untreated group A *Streptococcus* infection. ARF rates peak among children 5–14 years of age (*1*). ARF may permanently damage cardiac valves, producing chronic rheumatic heart disease (RHD), a serious, sometimes fatal, condition that may require surgery (*2*). Approximately half the children who experience an initial episode of ARF sustain cardiac damage, which persists as RHD for ≈15%–50% (*3*). Repeated ARF attacks (recurrent ARF) can produce new, and worsen existing, cardiac damage. If long-term prophylaxis (intramuscular injections of benzathine penicillin G [BPG]) is not administered regularly, ≈50% of ARF patients will experience recurrent ARF (*4*). Secondary prophylaxis is complicated by access to healthcare, cultural appropriateness of care delivery, injection-related discomfort, and health literacy. RHD can also develop without any previously recognized ARF (*5*,*6*). The World Health Organization recommends establishing patient registers to assist with best-practice patient management in areas where ARF persists. New Zealand lacks a national ARF register, despite a significant disease burden (*7*). 

In most high-income countries, ARF is rare; rates declined sharply from the 1960s. This decline is largely attributed to improved socioeconomic and living conditions that reduce group A *Streptococcus* infections and to increased use of antimicrobial drugs to treat infections before ARF onset (*1*,*8*–*10*). Pacific Islanders make up 7% of the New Zealand population; migration between New Zealand and other Pacific Island countries occurs regularly (*11*). ARF rates for indigenous Australian, New Zealand Māori, and Pacific Islander populations are among the highest in the world (*12*,*13*). In New Zealand, deaths from ARF are uncommon, but RHD causes ≈140 deaths and 600 hospitalizations annually; Māori and Pacific Islander persons are overrepresented (*14*). 

In New Zealand, the National Health Index number (NHI), a unique identifier, can identify and link a person’s information across health datasets. However, information regarding the extent to which ARF patients experience poor health outcomes is limited (*15*). Patient register data (which includes echocardiographic records) from Northern Territory, Australia, show that RHD developed within 10 years of a new ARF diagnosis for 61% of indigenous patients (*16*). In New Zealand, ARF is legally notifiable; however, considerable historic undernotification impairs the usefulness of surveillance data. Thus, epidemiologic analyses often rely on hospital admission data in the national minimum dataset (NMDS), which contains data on all publicly funded hospitalizations. The NDMS is affected by misdiagnosis and miscoding and is estimated to be 80% sensitive for detecting true ARF patients (*17*). NMDS specificity for identifying RHD is also an issue. Historically, patients who have valve disease without known cause were assigned International Classification of Diseases (ICD) codes for RHD (*18*). Analyses of ICD codes for RHD can thus overestimate true cases, particularly in high-income countries, where as few as 32% of patients assigned RHD codes have genuine probable/possible RHD (*19*). The ARF diagnosis can be complex and easy for clinicians to miss (*2*). Mild-to-moderate RHD may not necessitate hospital admission, and outpatient records are not compiled on a national level. Although it is recommended that persons with initial or recurrent ARF are hospitalized for optimal management (*2*), adult patients with minimal or no symptoms are often reluctant to be admitted. These issues make evaluating ARF prevention and control activities challenging (*7*).

The prognosis for patients with subclinical RHD is unclear. These patients may not experience clinically apparent ARF but rather experience cardiac changes consistent with RHD, detectable using echocardiography only. Without prophylaxis, some patients may experience further cardiac damage eventually resulting in clinically evident RHD. Therefore, echocardiographic screening of high-risk children to identify subclinical RHD cases and provide prophylactic treatment/monitoring may be needed to effectively reduce the RHD burden (*20*,*21*).

Given the absence of a national patient register from which to monitor New Zealand ARF patient outcomes, our first aim was to quantify the proportion of patients with initial ARF who progressed to hospitalization with recurrent ARF or RHD or died from circulatory causes (circulatory death) and to investigate their risk for disease progression according to selected demographic and clinical characteristics. Our second aim was to determine the proportion of patients with initial RHD who were hospitalized with previous ARF. Ethics approval was provided by the University of Otago Human Ethics Committee (HD 17/452), including a waiver of consent to use deidentified health data.

## Methods

### Aim 1: Determining Progression of Initial ARF to Recurrent ARF, RHD Hospitalization, and Early Death

In New Zealand, NMDS data with universal use of the NHI are available from 1988 on (*22*).; we extracted hospital admission data for 1989–2015. We extracted mortality data for 1989–2015 from the national Mortality Collection, which classifies the underlying cause of death for all registered deaths (*23*). We excluded from analysis non–New Zealand residents and all hospital transfers. 

#### RHD Dataset

**Figure 1:**
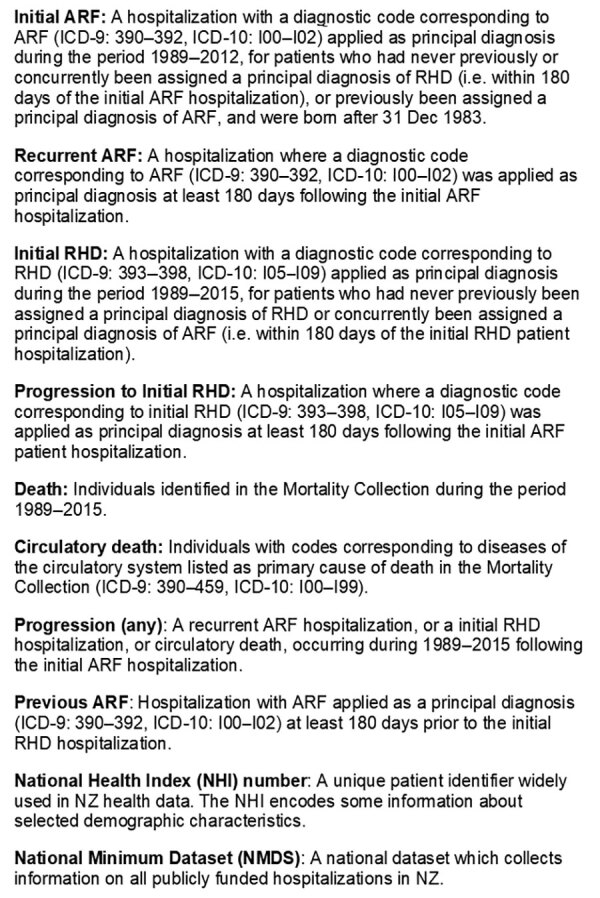
Definitions of terms used in study of ethnically disparate disease progression and outcomes among acute rheumatic fever patients in New Zealand, 1989–2015.

 We extracted NMDS data for patients hospitalized with RHD for the first time during 1989–2015 (Figure 1, Initial RHD). These patients had not previously received a diagnosis of RHD or a concurrent diagnosis of ARF.

#### Initial ARF Dataset

We extracted NMDS data for patients who were hospitalized and assigned a principal diagnosis of ARF during 1989–2012 (Figure 1, Initial ARF). To maximize data accuracy and completeness, we excluded patients born before January 1, 1984. Included patients would therefore have been <5 years of age at the start of the study period. Because ARF is very rare in children <4 years of age, all ARF hospitalizations would be captured in this cohort (*24*,*25*). To increase the average follow-up time, we excluded patients hospitalized for initial ARF after December 31, 2012. Consequently, the oldest possible participant age by the end of the follow-up period (December 31, 2015) was 31 years and the youngest possible age was 3 years.

**Figure 2:**
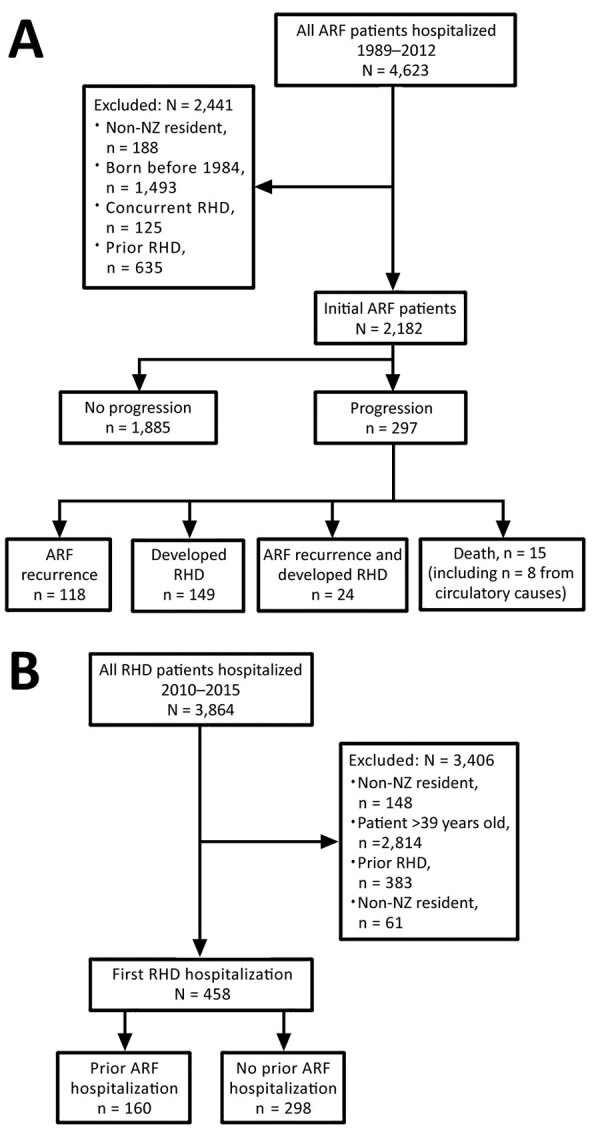
Progression of ARF and RHD among acute rheumatic fever patients in New Zealand, 1989–2015. A) Identification of patients with initial ARF and disease progression. B) Identification of patients with initial RHD and previous ARF. ARF, acute rheumatic fever; RHD, rheumatic heart disease.

We excluded persons who had concurrent RHD and initial ARF (Figure 2, panel A). Concurrent cases were identified when an encrypted NHI corresponding to an initial ARF hospitalization was matched to the RHD dataset and both hospitalizations occurred within 180 days of each other. The 180-day cutoff point was selected by using clinical advice from a pediatric cardiologist experienced in treating ARF and RHD. A data subset of initial ARF patients was created, as was a data subset of concurrent cases. Patients were considered to have had carditis if ICD codes 101, 102, 1020, 391, 392, or 3920 were listed with their initial ARF hospitalization.

#### Recurrent ARF Dataset

The encrypted NHI identified all repeated hospitalizations occurring within 180 days of each other for which ARF was the principal diagnosis during 1989–2015 (Figure 1, Recurrent ARF). A data subset for patients with recurrent ARF was created (Figure 2, panel A).

#### RHD Progression Dataset

We used the encrypted NHI to match persons in the initial ARF dataset with the RHD dataset (Figure 1, Progression to Initial RHD). We created a data subset of patients with initial ARF that progressed to hospitalization for RHD (Figure 2, panel A).

#### ARF Mortality Datasets

We used the encrypted NHI to match the initial ARF dataset with the Mortality Collection. When a match was made, we extracted the date and cause of death. We identified initial ARF patients who died before January 1, 2016. We noted when the primary cause of death was attributed to diseases of the circulatory system (Figure 1, Circulatory Death; codes 390–459 from ICD 9th Revision, 100–199 ICD 10th Revision). We created a data subset of initial ARF patients who died from circulatory causes (Figure 2, panel A).

#### Any Progression Dataset

We combined data subsets of patients with initial ARF who progressed to hospitalization with recurrent ARF or RHD, to circulatory death, or both (Figure 1, Progression [Any]). The resulting dataset identified initial ARF patients who experienced disease progression before January 1, 2016. We tabulated key demographic and clinical characteristics of patients who did and did not progress.

### Aim 2: Determining Proportion of RHD Patients with Previous ARF

Initial RHD patients were identified in NMDS data when an ICD code corresponding to RHD (Figure 1, Initial RHD) was applied for the first time as a principal diagnosis during January 1, 2010–December 31, 2015. To maximize chances of detecting the first hospitalization with ARF/RHD as a primary diagnosis, we excluded RHD patients >39 years of age. We applied inclusion and exclusion criteria when identifying initial RHD patients who had and had not been hospitalized with previous ARF (Figure 2, panel B).

We used the 180-day separation to distinguish ARF progression from patients who concurrently had ARF and RHD (aim 1) and from patients with multiple ARF hospitalizations for their first ARF episode (aim 2). When observing ARF progression, patients with ICD code(s) corresponding to initial RHD as principal diagnosis <180 days from their initial ARF hospitalization were classified as having concurrent ARF and RHD (aim 1). When RHD preceded ARF, patients with diagnostic code(s) corresponding to ARF applied as principal diagnosis <180 days of their initial RHD hospitalization were classified as having concurrent ARF and RHD (aim 2).

### Statistical Analyses

We used R software version 3.1.0 throughout our analysis (*26*). Demographic data analyzed included patient age at hospitalization, New Zealand resident status, sex, prioritized ethnicity, and 2006/2013 New Zealand Deprivation Index (NZDep06/NZDep13), all of which were encoded by the NHI. Prioritized ethnicity identifies persons belonging to multiple ethnic groups and reallocates a single ethnic group by using a prioritized order of Māori, Pacific Islander, Asian, and other (*27*). The NZDep06/NZDep13 classification system measures socioeconomic deprivation in small geographic areas by using census data (*28*). Quintile 1 represents persons living in the least deprived neighborhoods; quintile 5, the most deprived neighborhoods.

To investigate whether reported proportions differed significantly between groups, we used the χ^2^ test. We used the Mann-Whitney U test to compare differences in progression time from initial ARF hospitalization to RHD progression (aim 1) and time from preceding ARF to initial RHD hospitalization (aim 2). We used Kaplan-Meier modeling to estimate the probability of disease progression over a theoretical 9,791-day (i.e., 26.8-year) follow-up period by extrapolating observed progression rates. This period was the maximum time that any person in the dataset was observed. Outcomes were investigated individually and together as the “any progression” group. Observations were right censored at the end of the study period.

Generalized linear models calculated odds ratios (ORs) and 95% CIs of progression outcomes by selected characteristics. Cox-proportional hazard ratios (HRs) and 95% CIs described whether initial ARF patients with certain characteristics tended to experience disease progression sooner than others. We considered p<0.05 to be significant.

## Results

### Aim 1: Study Population

During 1989–2012, a total of 4,623 ARF patients were hospitalized with ARF for the first time; 2,182 met the inclusion criteria (Figure 2, panel A). The median follow-up time for this cohort was 10.4 years (range 3.0–26.8 years, interquartile range [IRQ] 6.4–15.3 years). Most initial ARF patients were 5–14 years of age (83.3%), male (57.9%), and of Māori (54.4%) or Pacific Islander (36.4%) ethnicity. Most (66.9%) were from NZDep06 quintile 5 (the most deprived neighborhoods). Just over half (51.9%) had carditis (Table 1).

**Table 1:** Key demographic and clinical characteristics of initial ARF patients born after December 31, 1983, and hospitalized during 1989–2012, New Zealand, outcomes through December 31, 2015*

Patient characteristics	All initial ARF patients, no.	No. (%) patients					
		Did not experience ARF progression	Experienced ARF progression	Recurrent ARF hospitalization	RHD hospitalization	Died from any cause	Died from circulatory causes
Total	2,182	1,885 (86.4)	297 (13.6)	142 (6.5)	173 (7.9)	15 (0.7)	8 (0.4)
Age group, y							
0–4	77	70 (90.9)	7 (9.1)	4 (5.2)	4 (5.2)	1 (1.3)	0
5–9	798	694 (87.0)	104 (13.0)	56 (7.0)	55 (6.9)	2 (0.3)	2 (0.3)
10–14	1,019	871 (85.5)	148 (14.5)	63 (6.2)	95 (9.3)	1 (0.1)	0
15–19	201	174 (86.6)	27 (13.4)	14 (7.0)	13 (6.5)	6 (3.0)	1 (0.5)
20–29	87	76 (87.4)	11 (12.6)	5 (5.7)	6 (6.9)	5 (5.7)	5 (5.7)
Sex							
M	1,264	1,123 (88.8)	141 (11.2)	72 (5.7)	78 (6.2)	10 (0.8)	5 (0.4)
F	918	762(83.0	156 (17.0)	70 (7.6)	95 (10.3)	5 (0.5)	3 (0.3)
Ethnicity (prioritized)							
Māori	1,189	1,025 (86.2)	164 (13.8)	80 (6.7)	97 (8.2)	8 (0.7)	4 (0.3)
Pacific Islander	795	681 (85.7)	114 (14.3	50 (6.3)	68 (8.6)	6 (0.8)	4 (0.5
European/other	198	179 (90.4)	19 (9.6)	12 6.1)	8 (4.0)	1 (0.5)	0
NZDep06 quintile							
1	68	59 (86.8)	9 (13.2)	5 (7.4)	9 (13.2)	0	0
2	102	88 (86.3)	14 (13.7)	6 (5.9)	5 (4.9)	0	0
3	187	155 (82.9)	32 (17.1)	11 (5.9)	12 (6.4)	2 (1.1)	2 (1.1)
4	353	315 (89.2)	38 (10.8)	19 (5.4)	22 (6.2)	3 (0.8)	1 (0.3)
5	14,60	1,259 (86.2)	201 (13.8)	99 (6.8)	124 (8.5)	10 (0.7)	5 (0.3)
Unknown	12	9 (75.0)	3 (25.0)	2 (16.7)	1 (8.3)	0	0
Carditis							
No	1,050	951 (90.6)	99 (9.4)	59 (5.6)	48 (4.6)	5 (0.5)	1 (0.1)
Yes	1,132	934 (82.5)	198 (17.5)	83 (7.3)	125 (11.0)	10 (0.9)	7 (0.6)

*ARF, acute rheumatic fever; NZDep06 index, 2006 New Zealand Deprivation Index; RHD, rheumatic heart disease.

Of the initial ARF patients hospitalized for RHD, 42% (125/298) had RHD concurrently and were excluded (aim 1). Similarly, of the initial RHD patients who experienced ARF, 46% (65/142) had concurrent ARF (aim 2) and were excluded. The time distribution to progression supports use of the 180-day cutoff (Appendix Figure 1, panels A, B).

#### Aim 1A: Risk for Progression from Initial ARF to Recurrent ARF or RHD Hospitalization and Early Death

A total of 297 (13.6%) of the 2,182 patients with initial ARF experienced disease progression before January 1, 2016. Of these, 142 (6.5%) were hospitalized with recurrent ARF and 173 (7.9%) with RHD; 24 were hospitalized for both. Fifteen initial ARF patients died, 8 from circulatory causes (Figure 2, panel A).

The median time from initial ARF to recurrent ARF hospitalization was 3.2 years (IQR 1.9–8.4 years) and to RHD hospitalization was 4.0 years (IQR 1.9–8.4 years). The median time to circulatory death was 10.4 years (IQR 3.3–12.8 years).

**Figure 3:**
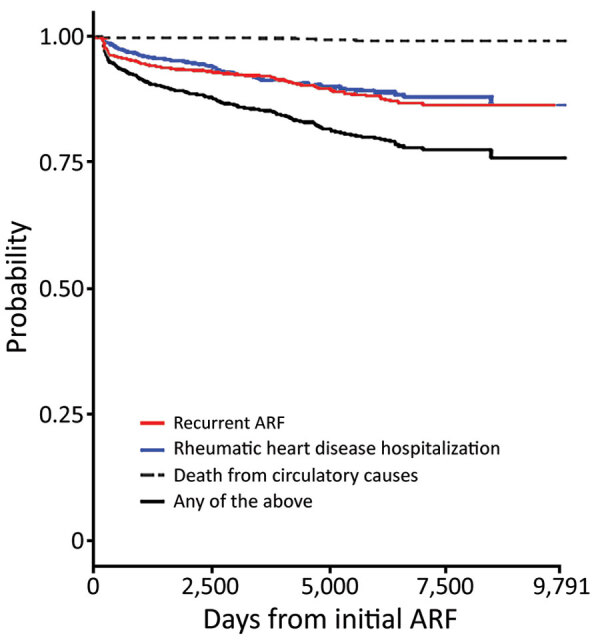
Probability of disease progression to recurrent ARF, hospitalization for rheumatic heart disease, or circulatory death after hospitalization for initial ARF >9,791 days among acute rheumatic fever patients in New Zealand, 1989–2015. ARF, acute rheumatic fever.

The overall probability of experiencing disease progression (to hospitalization with recurrent ARF/RHD or to circulatory death) within a theoretical 9,791 days from the initial ARF hospitalization was 24.0%. When progression outcomes were considered individually, the probability of recurrent ARF hospitalization was 23.5%, as was the probability of being hospitalized for RHD. The risk for death was low: 1.0% (Figure 3).

#### Aim 1B: Risk Factors for Progression from Initial ARF 

Risk for disease progression was higher for Māori (OR 1.68, 95% CI 1.10–2.67) and Pacific Islander (OR 2.12, 95% CI 1.37–3.39) patients than for persons of European or other ethnicities. Progression occurred sooner for Māori (HR 1.89, 95% CI 1.24–2.88) and Pacific Islander (HR 2.35, 95% CI 1.54–3.60) patients. Disease progression was twice as likely for patients with carditis (OR 2.00, 95% CI 1.57–2.54) than without carditis, and progression occurred sooner (HR 1.94, 95% CI 1.55–2.43). We noted no significant differences in risk for disease progression by sex, age, or NZDep 06 quintile (Table 2). No factors in Table 2 were found to be significant predictors of recurrent ARF.

**Table 2:** Factors influencing the likelihood of disease progression to recurrent ARF, hospitalization for RHD, or circulatory death after hospitalization for initial ARF, New Zealand, 1989–2015*

Factor	Patient progression from initial ARF	
	Cox-proportional model, HR (95% CI)	Generalized linear model, OR (95% CI)
Age group, y		
<5	0.59 (0.24–1.4)	1.17 (0.45–3.01)
5–9	0.88 (0.46–1.7)	1.43 (0.75–3.01)
10–14	1.02 (0.54–1.9)	1.54 (0.82–3.23)
15–19	1.02 (0.50–2.1)	1.30 (0.62–2.92)
20–29	Referent	Referent
Sex		
F	1.01 (0.81–1.25)	1.02 (0.80–1.28)
M	Referent	Referent
Ethnicity		
Māori	**1.89 (1.24–2.88)**	**1.68 (1.10–2.67)**
Pacific Islander	**2.35 (1.54–3.60)**	**2.12 (1.37–3.39)**
European/other	Referent	Referent
NZDep06 quintile		
1	Referent	Referent
2	0.56 (0.25–1.23)	0.47 (0.19–1.09)
3	0.88 (0.47–1.66)	0.77 (0.39–1.58)
4	0.70 (0.38–1.27)	0.61 (0.32–1.21)
5	0.88 (0.51–1.50)	0.76 (0.42–1.43)
ARF diagnostic code denoting carditis		
Yes	**1.94 (1.55–2.43)**	**2.00 (1.57–2.54)**
No	Referent	Referent

*Boldface indicates statistical significance. ARF, acute rheumatic fever; HR, hazard ratio; NZDep06 index, 2006 New Zealand Deprivation Index; OR, odds ratio; RHD, rheumatic heart disease.

Risk for disease progression to RHD hospitalization was higher for Māori (OR 2.09, 95% CI 1.09–4.52) and Pacific Islander (OR 3.64, 95% CI 1.91–7.86) patients than for patients of European or other ethnicities and occurred sooner (HR 2.54, 95% CI 1.27–5.10 for Māori; HR 2.53, 95% CI 1.27–5.05 for Pacific Islanders). Initial ARF patients with carditis were more likely to experience RHD (OR 5.19, 95% CI 3.52–7.89) than those without carditis. Patients with initial ARF whose condition progressed to recurrent ARF were more likely to experience progression to hospitalization for RHD than patients who did not experience recurrent ARF (OR 3.10, 95% CI 2.07-4.55). Small patient numbers meant that no factors predicted circulatory death, with the exception of carditis (OR 6.52, 95% CI 1.16–122.00; Appendix Table 2).

### Aim 2: Proportion of Initial RHD Patients with Preceding ARF

A total of 3,836 patients were hospitalized with RHD during 2010–2015; of these, 435 patients with initial RHD met the inclusion criteria (Figure 2, panel B), 102 of whom were also included in the initial ARF dataset for aim 1. Most patients were female (229, 52.6%), Pacific Islander (207, 47.6%), or Māori (176, 40.5%) and were from the most deprived neighborhoods (271 [62.3%] NZDep13 quintile 5). Previous hospitalization for ARF (i.e., >180 days before initial RHD hospitalization) was detected for 77 patients (17.8%; Figure 2, panel B). Of the 335 initial RHD patients <30 years of age, 19.4% had been previously hospitalized for ARF.

Of the Māori patients, 21.6% were previously hospitalized for ARF, as were 18.4% of Pacific Islander patients. A significantly lower proportion (1.9%) of patients of European and other ethnicities were previously hospitalized for ARF (p<2.2 × 10^–16^). A lower proportion of female patients (11.4%) were previously hospitalized for ARF than were male patients (24.8%; p = 2.565 × 10^–6^). Of the patients from NZDep quintile 5, a total of 19.9% were previously hospitalized for ARF, as were 15.4% of patients from other quintiles (p = 0.048; Appendix Table 1). There was no difference in overall time of progression from initial ARF to RHD hospitalization compared with time from initial RHD going back to preceding ARF hospitalization (p˃0.05; Appendix Figure 1).

## Discussion

This study demonstrates concerning ethnic inequities in ARF progression. By the end of the study period, 14% of initial ARF patients (with no concurrent RHD) had experienced progression to recurrent ARF, RHD, or circulatory death. However, ARF progression was approximately twice as likely for Māori and Pacific Islander patients and occurred approximately twice more rapidly. It is concerning that of 435 initial RHD patients <40 years of age, <1 in 5 were hospitalized with preceding ARF, severely limiting opportunities for secondary prevention. Ethnic inequities in ARF progression add to extreme ethnic inequities in the burden of ARF (*24*,*25*). Possible reasons for increased illness among Māori and Pacific Islander patients include the inequitable distribution of the underlying determinants of health, such as access to health services, nutrition, and a healthy home environment (*29*,*30*). Genetic and immunologic factors may also contribute (*30*–*33*). Similar findings have been reported for indigenous patients in Australia (*15*).

That 14% of the initial ARF cohort experienced progression suggests failures in secondary prophylaxis to which Māori and Pacific Islander patients may experience barriers. We support creating a national patient register by drawing on data from regional registers. The goal would be to improve secondary prophylaxis uptake by coordinating treatment for patients who are frequently mobile. There is widespread support for a national register among stakeholders, which could be expanded to monitor patients’ RHD status and health outcomes (*7*,*34*). Previous attempts to set up a national register have failed because of privacy concerns. A perceived lack of political will to implement such a register has been noted (*35*,*36*).

Disease progression for New Zealand ARF patients overall seems to be considerably less than that reported for indigenous patients in Australia (*16*). This difference probably reflects multiple factors, including difficulty delivering consistent medical treatment in remote areas and use of echocardiography outreach clinics in Australia (which may detect RHD sooner than when signs/symptoms otherwise come to medical attention) (*16*,*37*,*38*). Our findings may be specific to New Zealand. 

Although we did not identify differences in ARF progression by sex, these differences have been reported elsewhere (*16*,*39*). Our finding that 12% of female patients with initial RHD were previously hospitalized with ARF, compared with 25% of male patients, may suggest that clinical manifestations and outcomes for female patients warrant investigation.

More than 80% of young initial RHD patients had not been previously hospitalized for ARF, which indicates that many ARF patients do not come to clinical attention and miss prophylactic treatment. Increasing public and clinician awareness of ARF, echocardiographic screening for high-risk children, and new diagnostic tools may improve case identification (*21*,*40*). If a clear prognostic benefit is demonstrated from echocardiography screening programs, this finding may strongly support the use of targeted screening among high-risk New Zealand children. It is unlikely that RHD detected through echocardiography screening studies would have affected this analysis because they would receive outpatient assessment (*41*).

The reliance on hospital data is a major limitation of, and justification for, this study. In New Zealand, gaps in data completeness for ARF/RHD are closing; however, the study period is affected (*42*). Some patients may have been inappropriately included or missed from this analysis, or progression outcomes may be misclassified. This study markedly underestimates the proportion of ARF patients whose condition will ultimately progress because of limited follow-up time; furthermore, hospitalization data do not capture outpatients (*2*). Although a prospective study design would enable a more nuanced analysis of ARF progression, severe outcomes may take many years to develop (*43*). Repeat analyses of this study cohort will provide a more complete assessment of progression risk. Migration of RHD patients into New Zealand may account for some occurrences where no preceding ARF hospitalization was identified (*44*). The high (94%) proportion of children <10 years of age with initial RHD and no preceding/concurrent ARF hospitalization may result from miscoding (with ARF incorrectly coded as RHD; Appendix Table 1). Use of the >180-day window was supported when examining time intervals to progression (Appendix) and by the small number of studies reporting on ARF progression (*45*,*46*). A quality systematic patient audit would be valuable for assessing the validity of diagnostic coding. It would also be useful to audit a sample of initial RHD patients not previously hospitalized for ARF to see if diagnostic opportunities had been missed. 

A key study strength is use of the encrypted NHI to identify persons within and between datasets, which permits inclusion of an entire national cohort. Hospitalization is the standard of care for all patients with suspected initial ARF in New Zealand (*2*). Ambulatory care data and prophylaxis data were not available (*2*). Published data on BPG adherence are inconsistently available. A regional study of 77 ARF patients identified 51% as fully adherent to BPG prophylaxis (*47*). An audit from Auckland (where ≈50% of patients reside) indicated that ≈96% of ARF patients were fully adherent in the 2 largest regions, but adherence fell from 93% in 1998 to 86% in 2000 in the smaller (Waitemata) region (*48*). The extent to which progression rates were affected by ARF patients’ adherence to secondary prophylaxis in this analysis is unknown.

In summary, our study better defines ARF disease progression in New Zealand. After their initial ARF hospitalization, 14% of patients were hospitalized with recurrent ARF or RHD or died; that proportion will probably increase over time. Māori and Pacific Islander patients face an increased risk for ARF progression. Four fifths of initial RHD patients had no preceding ARF hospitalization recorded, thus limiting opportunities for prophylaxis. The need to enhance clinical care delivery for underserved groups is strongly indicated. A national patient register may improve prophylaxis uptake, clinical service coordination, and sector performance monitoring. Further research into echocardiography screening is needed. Our results show a clear need to address the major modifiable determinants of health and equity; ARF and RHD represent indicators of progress that should be closely monitored.

Appendix Supplementary data from study of ethnically disparate disease progression and outcomes among acute rheumatic fever patients in New Zealand, 1989–2015.
