# Supplementary material for: Ethnically Disparate Disease Progression and Outcomes among Acute Rheumatic Fever Patients in New Zealand, 1989–2015
Source: Emerg Infect Dis. 2021 Jul;27(7):1893–902. doi: 10.3201/eid2707.203045 (PMC8237904; doi:10.3201/eid2707.203045)
Supplement: Appendix — Supplementary data from study of ethnically disparate disease progression and outcomes among acute rheumatic fever patients in New Zealand, 1989–2015. [file 20-3045-Techapp-s1.pdf]

Article DOI: https://doi.org/10.3201/eid2707.203045

Ethnically Disparate Disease Progression and Outcomes among Acute Rheumatic Fever Patients in New Zealand, 1989–2015

Appendix

Appendix Table 1. Initial RHD patients aged <40 years old diagnosed 2010-2015, with and without preceding ARF hospitalization*

	Total Initial RHD patients	Initial RHD patients with preceding Initial ARF hospitalization (Initial ARF ≥ 180 days)		Initial RHD patients with no preceding Initial ARF hospitalization (Initial ARF ≥ 180 days)	
Patient characteristic	Number (N)	Number (N)	Proportion of patients (%)	Number (N)	Proportion of total RHD patients (%)
Total	435	77	17.8	358	82.2
Age group at RHD diagnosis (years)					
<5	11	0	0.0	11	100.0
5-9	72	5	6.9	67	93.1
10-14	111	16	14.4	95	85.6
15-19	55	21	38.2	34	61.8
20-29	86	23	26.7	63	73.3
	100	12	12.0	88	88.0
Gender					
Female	229	26	11.4	203	88.6
Male	206	51	24.8	155	75.2
Ethnicity					
Māori	176	38	21.6	138	78.4
Pacific	207	38	18.4	169	81.6
European/other	52	1	1.9	51	98.1
NZDep06 Index					
Quintile 1	11	1	9.1	10	90.9
Quintile 2	17	2	11.8	15	88.2
Quintile 3	38	5	13.2	33	86.8
Quintile 4	77	14	18.2	63	81.8
Quintile 5	271	54	19.9	217	80.1
Unknown	21	1	4.8	20	95.2
Aged <30 years when first diagnosed with RHD	335	65	19.4	270	88.6

*RHD, rheumatic heart disease; ARF, acute rheumatic fever.

Appendix Table 2. Factors influencing the likelihood of individual progression outcome (Recurrent ARF/RHD hospitalization/circulatory death) following initial ARF diagnosis*

Factor	HR (95% CI) of initial ARF patient progression (Cox-proportional model)	OR (95% CI) of initial ARF patient progression (Generalised linear model)
Progression outcome: Recurrent ARF		
Age group (years)		
<5	0.53 (0.18-1.58)	0.98 (0.30-3.08)
5-9	0.76 (0.35-1.65)	1.17 (0.56-2.87)
10-14	0.83 (0.38-1.80)	1.18 (0.56-2.87)
15-19	0.78 (0.32-1.91)	0.92 (0.37-2.49)
20-29	Reference	Reference
Gender		
Female	1.08 (0.81-1.43)	1.10 (0.82-1.47)
Male	Reference	Reference
Ethnicity		
Maori	1.40 (0.88-2.24)	1.22 (0.76-2.03)
Pacific	1.20 (0.73-1.96)	1.02 (0.62-1.74)
European/Other	Reference	Reference
NZDep06 Index		

Factor	HR (95% CI) of initial ARF patient progression (Cox-proportional model)	OR (95% CI) of initial ARF patient progression (Generalised linear model)
Quintile 1	Reference	Reference
Quintile 2	0.72 (0.27-1.92)	0.64 (0.22-1.82)
Quintile 3	0.79 (0.34-1.86)	0.70 (0.29-1.81)
Quintile 4	0.71 (0.33-1.57)	0.65 (0.29-1.58)
Quintile 5	0.87 (0.43-1.78)	0.77 (0.38-1.78)
ARF diagnostic code denoting carditis		
Yes	1.03 (0.78-1.36)	0.99 (0.74-1.33)
No	Reference	Reference
Progression outcome: RHD Hospitalisation		
Age group (years)		
<5	**0.23 (0.05-0.99)**	1.97 (0.47-9.88)
5-9	**0.29 (0.09-0.95)**	2.40 (0.86-9.97)
10-14	**0.30 (0.09-0.96)**	2.85 (1.04-11.75)
15-19	0.39 (0.11-1.34)	2.26 (0.72-9.94)
20-29	Reference	Reference
Gender		
Female	1.10 (0.82-1.49)	0.96 (0.70-1.30)
Male	Reference	Reference
Ethnicity		
Maori	**2.54 (1.27- 5.10)**	**2.09 (1.09-4.52)**
Pacific	**2.53 (1.27-5.05)**	**3.64 (1.91-7.86)**
European/Other	Reference	Reference
NZDep06 Index		
Quintile 1	Reference	Reference
Quintile 2	1.81 (0.45-7.33)	**0.26 (0.06-0.99)**
Quintile 3	1.61 (0.63-4.14)	0.87 (0.36-2.35)
Quintile 4	2.16 (0.88-5.33)	0.72 (0.32-1.87)
Quintile 5	1.96 (0.85-4.49)	0.82 (0.39-1.99)
ARF diagnostic code denoting carditis		
Yes	1.08 (0.73-1.60)	**5.19 (3.52-7.89)**
No	Reference	Reference
ARF recurrence		
Yes	**0.66 (0.46-0.95)**	**3.10 (2.07-4.55)**
No	Reference	Reference
Progression outcome: Circulatory death		
Age group (years)		
<5	Not calculable	Not calculable
5-9	Not calculable	Not calculable
10-14	Not calculable	Not calculable
15-19	Not calculable	Not calculable
20-29	Reference	Reference
Gender		
Female	0.81 (0.19-3.38)	0.82 (0.17-3.37)
Male	Reference	Reference
Ethnicity		
Maori	Not calculable	Not calculable
Pacific	Not calculable	Not calculable
European/Other	Reference	Reference
NZDep06 Index		
Quintile 1	Reference	Reference
Quintile 2	Not calculable	Not calculable
Quintile 3	Not calculable	Not calculable
Quintile 4	Not calculable	Not calculable
Quintile 5	Not calculable	Not calculable
ARF diagnostic code denoting carditis		
Yes	6.96 (0.86-56.62)	**6.52 (1.16-122.00)**
No	Reference	Reference
ARF recurrence		
Yes	1.18 (0.15- 9.63)	1.44 (0.08-8.16)
No	Reference	Reference

*HR, hazard ratio; OR, odds ratio; CI, confidence interval; ARF, acute rheumatic fever. Bold typeface indicates statistical significance.

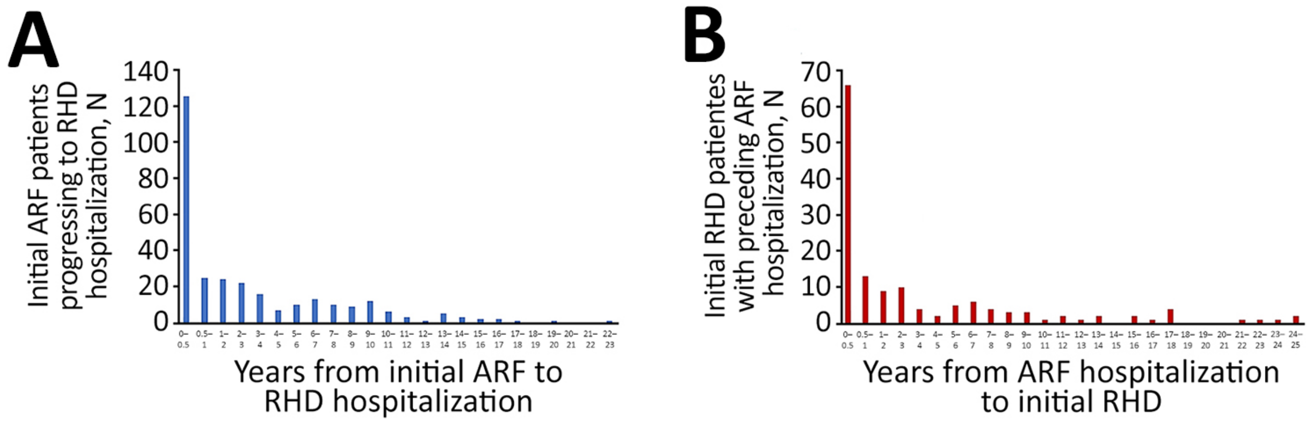

A total of 125 concurrent patients were diagnosed with RHD for the first time <180 days from their first ARF diagnosis. They are shown on histogram (A) in the 0-0.5 year category. These concurrent patients are excluded from calculations of progression risk and progression time.

Median time from initial ARF to initial RHD progression: 4.0 years, range: 0.5-22.1, IRQ: 1.9-8.1 years.

A total of 65 concurrent patients had a first hospitalisation with ARF <180 days preceding their first RHD diagnosis. They are shown on histogram (B) in the 0-0.5 year category. These concurrent patients are excluded from calculations of progression risk and progression time.

Median time from preceding initial ARF hospitalisation to initial RHD: 5.2 years, range: 0.5-24.8 years, IRQ: 1.6-9.2 years.

These time intervals were similar (p=0.4, Mann Whitney U test)

NB: A total of 610 patients were re-hospitalised with ARF <180 days following their initial ARF hospitalisation. These repeated hospitalisations were excluded from calculations of recurrent ARF progression risk and progression time as they likely corresponded to complications occurring during the first ARF episode.

Appendix Figure. A) Years from initial ARF hospitalization to RHD progression hospitalization, B) Days from preceding ARF hospitalization to initial RHD.
